# Changes in malaria epidemiology in Germany, 2001–2016: a time series analysis

**DOI:** 10.1186/s12936-018-2175-y

**Published:** 2018-01-15

**Authors:** Sabine Vygen-Bonnet, Klaus Stark

**Affiliations:** Unit of Gastrointestinal Infections, Zoonoses and Tropical Infections, Department of Infectious Disease Epidemiology, Robert Koch-Institute, Seestr. 10, 13353 Berlin, Germany

**Keywords:** Imported malaria, *P. vivax*, *P. knowlesi*, Refugees, Eritrea, Germany, Chemoprophylaxis

## Abstract

**Background:**

German surveillance data showed a sharp rise of malaria cases in 2014 and 2015 due to the increased arrival of refugees from malaria endemic countries. A time series analysis of data from 2001 to 2016 was performed in order to describe the epidemiology of imported malaria in Germany in general and of the recent increase in particular.

**Results:**

In total, 11,678 malaria cases were notified between 2001 and 2016 (range 526–1063 cases/year). Newly arriving refugees averaged 10 cases/year (1.5%) in 2001–13 and 292.5 cases/year (28.3%) in 2014–15. *Plasmodium* (*P*.) *falciparum* was the most frequently reported species (range 57.2–85.8%), followed by *P. vivax* (range during 2001–2013: 7.6–18.1%; during 2014–2015, mean 31.3%). In 2014–15, 22.3% of all *P. vivax* cases were refugees from Eritrea and 3.3% from other countries of the Horn of Africa; in 2015 and 2016, 19.5% were refugees from Afghanistan and Pakistan. Five *P. knowlesi* malaria infections were reportedly acquired in Thailand between 2012 and 2016. Total numbers of malaria notifications among native Germans and residents with migration background showed an increasing trend since 2007. Chemoprophylaxis use was reported for 24.3% (1695/6984) of cases and showed a declining trend. Native German cases took significantly more frequently chemoprophylaxis than cases with migration background (32.6% vs. 17.9%; p < 0.001).

**Discussion/conclusions:**

The steep rise in vivax malaria notifications in 2014 and 2015 was mainly due to newly arriving refugees from Eritrea but also from other countries of the Horn of Africa and South Asia. Clinicians should include malaria in their differential diagnosis in case of a febrile illness in the respective population and consider vivax malaria even if arrival to Germany dates back several months. Over the past 10 years, malaria notifications among native Germans and residents with migration background showed an increasing trend. Use of chemoprophylaxis was insufficient in both groups and deteriorating. New strategies need to be found to increase compliance to chemoprophylaxis recommendations. The surveillance provides valuable data for epidemiological assessment of imported malaria in Germany.

## Background

The number of malaria-endemic countries and territories has decreased since 2000 from 108 to 91 in 2016 [1]. The global malaria incidence rate was estimated to have fallen by 21% between 2010 and 2015. This success was largely attributed to widely deployed malaria control measures. The decline was most pronounced in Europe—Europe was declared malaria free in 2016 [2]—and South-East Asia and less marked in the African Region, where still 90% of malaria infections occur [1].

In Germany, notified malaria infections are generally imported. Autochthonous vector borne transmissions are extremely rare events due to the timely treatment of parasitaemic patients, the lack of highly competent vectors, and the unfavourable climatic conditions [3, 4]. Trends in malaria epidemiology in Germany depend on the number of travellers to, and immigrants from malaria-endemic countries, malaria endemicity at destination or home country, respectively, and the choices of the travellers regarding exposure prophylaxis and adherence to chemoprophylaxis recommendations. Laboratory malaria diagnosis is notifiable to the national public health institute [Robert Koch-Institute (RKI)] according to the German Infection Protection Act [5]. Malaria notification data are an important source for monitoring trends and provide information about the risk of travellers to different destinations and about the impact of pre-travel advice regarding chemoprophylaxis.

A sharp increase in malaria notifications occurred in 2014, coinciding with a large influx of newly arriving refugees in Germany. Roggelin et al. [6] reported an increase of vivax malaria in 2014–2015 in refugees from Eritrea admitted to the University Medical Centre in Hamburg [6, 7], as also described in Sweden in 2014 [8, Sonden et al., personal communication], and the Netherlands in 2017 [9].

In order to obtain a better understanding of malaria epidemiology in Germany in general and of the recent increase in malaria cases in particular, time series analysis using national notification data from the past 16 years was performed, aiming to describe demographic characteristics of cases, distribution of *Plasmodium* species, countries of infection, and geographic origin of cases over the time period between 2001 and 2016. Chemoprophylaxis use and disease outcomes were also analysed.

## Methods

Direct or indirect detection of any type of *Plasmodium* in human blood is notifiable by laboratories directly to RKI, in an anonymous manner, regardless of the applied laboratory method. The RKI sends standardized data forms to laboratories for this purpose. Clinical data are added by an attending physician, filling in a second reporting form, which is forwarded to him or her by the laboratory. Both reporting forms are matched together at national level and entered into a database. The form asks for information on month and year of birth, gender, the three first digits of the patient’s home post code, month and year of malaria diagnosis, *Plasmodium* species, geographic origin, if applicable date of immigration to Germany, travel history with dates, travel purpose and destinations, information on prophylaxis and treatment, hospitalization and death. An automated algorithm identifies potential double entries. These are manually screened and datasets are excluded if they prove to belong to the same patient. Laboratories or clinics are contacted by telephone or telefax in order to complete data.

### Case definition

Case definition includes any person who had a direct or indirect proof of malaria parasite from their blood and whose country of residence was Germany at the time of diagnosis.

### Data analysis

STATA SE14 and Microsoft Excel 2010 was used for performing time series analysis on notification data. Since 2001, when the German Infection Protection Act came into force, data was collected in a consistent manner, therefore data from 2001 to 2016 was included. The ‘year of notification’ was set as time variable. In order to be able to distinguish sub-groups among malaria cases according to their travel history and country of infection, four groups of different origin were defined:

Group 1: ‘native German cases’ were cases with country of origin indicated as Germany.
Group 2: ‘residents with migration background’ were cases whose country of origin was reported to be any other country than Germany and who were, not categorized as ‘recent refugees’ (group 3).
Group 3: ‘recent refugees’ were cases fulfilling the following criteria:
  - Person reported as being in Germany as ‘immigrant/asylum seeker/refugee’ or whose purpose of travel was indicated as ‘immigrant/asylum seeker/refugee’ or with no information of why being in Germany or purpose of travel AND who had arrived in Germany during 1 year prior to malaria diagnosis, OR.
  - In case of missing information on why being in Germany and purpose of travel and/or date of arrival in Germany, person whose country of origin was a country which was among the top 10 countries of the refugee statistics of the German Federal Office for Migration and Refugees (BAMF) for the respective year of malaria notification. As the date of arrival was often incomplete with missing month and day, only the year of arrival was used for calculating the 1-year time interval.
Group 4: cases not belonging to group 1–3, with incomplete or missing data concerning the criteria for group 1–3.

In order to calculate the percentage of cases who reported the use of chemoprophylaxis only cases with reported countries of infection, for which chemoprophylaxis was recommended for at least part of the country, based on the current malaria prophylaxis recommendations of the German society of tropical medicine and international health [10], were taken into account. Travel destinations for which in the past, but not any more in 2016, malaria prophylaxis was recommended were excluded from the calculations. The denominator included only cases with information on chemoprophylaxis use. Native German cases and cases with migration background were analysed separately. Recent refugees who acquired malaria in their home countries or on their travel route were not taken into account when looking at use of chemoprophylaxis.

If ‘mixed infection’ was indicated for *Plasmodium* species, the data collection form did not indicate the different species involved. Therefore, mixed infections were treated as ‘information on species missing’ and excluded when looking at the percentage of species and hospital admission rates according to different species.

Chi squared-test, t-test and Wilcoxon–Mann–Whitney-test were used in order to compare sub-groups. Differences were considered statistically significant at p < 0.05. Malaria incidence in refugees was calculated using data from BAMF [11] and malaria incidence in travellers for 2012–2016 using data on air travel departures to malaria-endemic countries, from the German Federal Statistical Office as denominators [12]. Recent refugees were excluded when calculating incidences of malaria based on air travel departure data.

## Results

### Data completeness

Overall, 11,678 malaria cases were included in the analysis, which were notified in Germany over 16 years (2001–2016). 618 cases not fulfilling the reference definition and 804 datasets, which were double notifications, were excluded from the analysis. Since 2001, reported malaria case numbers steadily declined from 1049 cases in 2001 to 542 in 2007. Subsequently they remained relatively stable until 2013, and rose in 2014–2016 by 43% compared to the mean of the previous 10 years. The lowest annual number of malaria cases was reported in 2009 (n = 526) and the highest in 2015 (n = 1063).

### Gender and age distribution and geographic origin of cases

During all years more male than female cases were observed. Between 2001 and 2013 male cases accounted for about two-thirds of cases (69.2%; 5878/8492). In 2014–2016, the male proportion rose to more than three-quarters of cases (75.7%; 2277/3008).

**Fig. 1:**
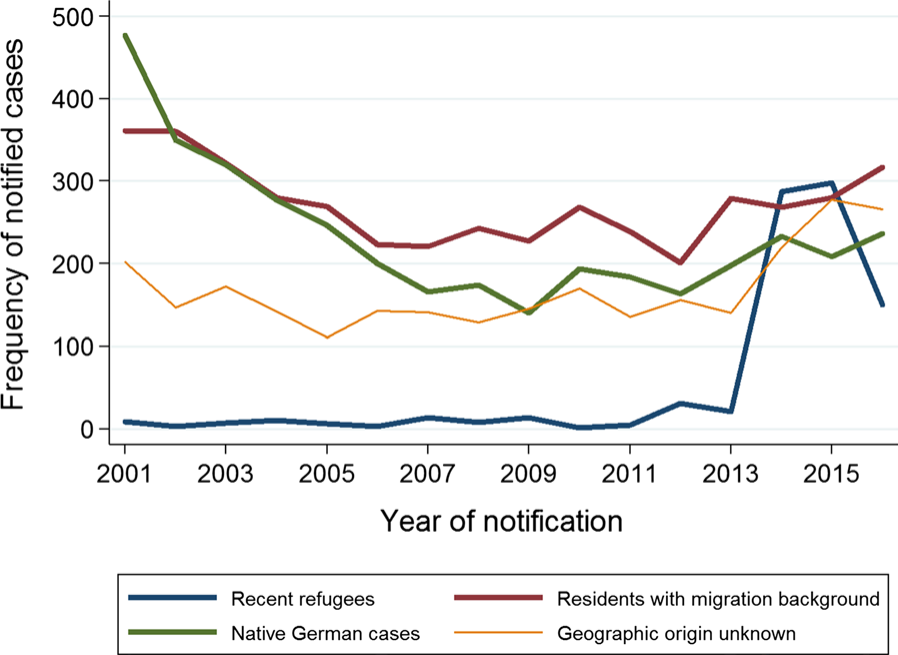
Malaria in Germany: frequency of case origin. Time series 2001–2016 (n = 11,678)

Native German cases were the largest group in 2001 (45.5%; 477/1049) (Fig. 1). The absolute number was declining until 2009 (26.6%; 140/526), and was then showing a rising trend (24.1%; 231/960) until 2016. From 2005, cases with migration background were the predominant group of malaria patients (except 2014 and 2015) with 316 annual cases in 2016 (32.9%; 316/960) and an overall rising trend since 2005 (42.6%; 269/632). From 2001 to 2013 on average 10.2 (1.5%; 10.2/665.2) cases/year were notified among newly arriving refugees. In 2014 and 2015 refugees accounted for a mean percentage of 28.3 (292.5/1035.5), declining to 15.5% (150/960) in 2016.

The percentage of children and adolescents (< 18 years) among all malaria cases notified in Germany was fairly stable during 2001–2013 with a mean of 9.7% (in average 64.3/665.2 cases/year were < 18 years old) and rose to 16.9% in 2014–16 (mean: 171/1010.3 cases/year). This increase was almost entirely due to the group of recent refugees, who were significantly younger than other cases (p < 0.001).

### *Plasmodium* species

**Fig. 2:**
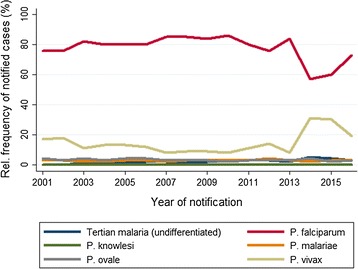
Malaria in Germany: relative frequency of malaria species. Time series 2001–2016 (n = 10,794)

**Fig. 3:**
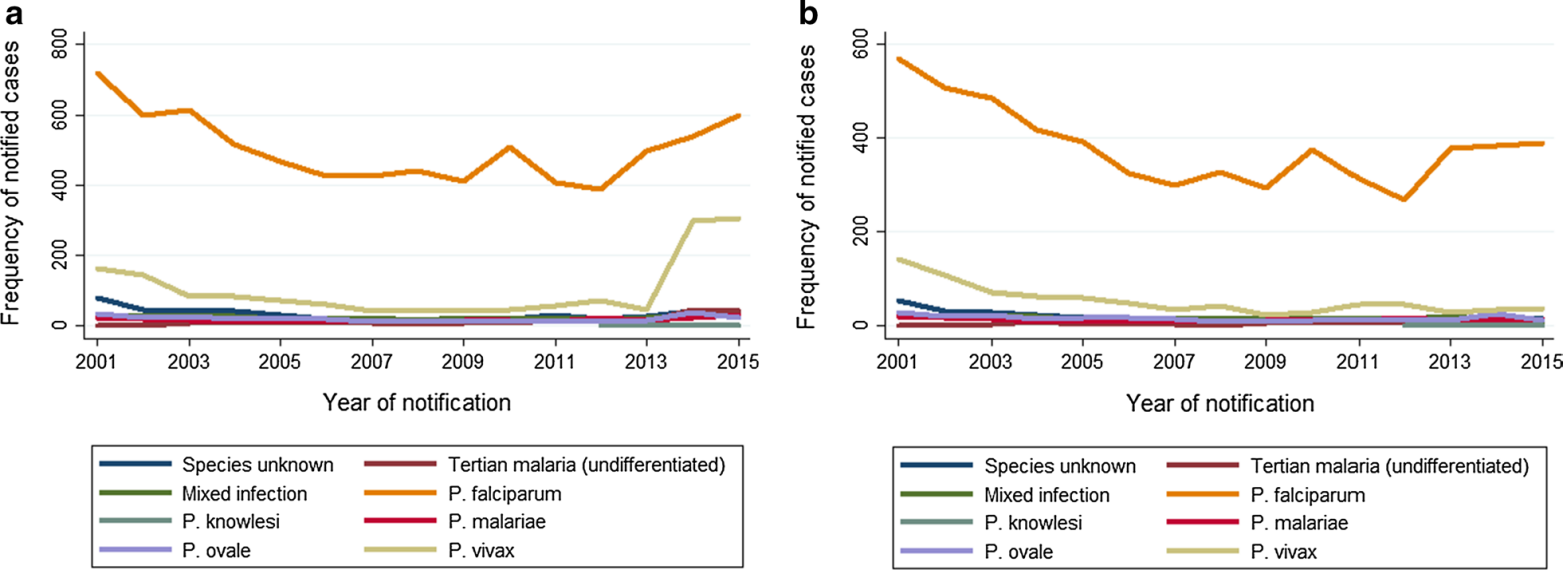
Malaria in Germany. **a** Frequency of malaria species overall (n = 11,168). **b** Frequency of malaria species after withdrawal of refugees and cases with unknown origin (n = 7827). Time series 2001–2016

During the entire observation period, *P. falciparum* was the most frequently reported species (Fig. 2), ranging from 57.2% (536/937) in 2014 to 85.8% (509/593) in 2010. *Plasmodium vivax* was the second most reported species during 2001–2013: between 7.6% (45/593) in 2010 and 18.1% (142/785) in 2002. In 2014 and 2015, numbers of vivax malaria rose to 31.3% (293/937) and 30.4% (305/1002), respectively, and came back down to 18.5% (168/907) in 2016. In 2014 and 2015, 22.3% (384/1724) of all *P. vivax* cases were refugees from Eritrea and 3.3% (20/598) from other countries in the Horn of Africa. In 2015 and 2016, 19.5% (92/473) were refugees from Afghanistan and Pakistan. When plotting species distribution against time after withdrawing recent refugees and cases with missing information on their origin, the rise of *P. vivax* cases in 2014 and 2015 was no longer visible (Fig. 3b). While the percentage of *P. falciparum* infections declined and *P. vivax* infections increased in 2014 and 2015, the absolute number of *P. falciparum* infections rose from 430 average annual cases in 2006–2012, to 516 in 2013–2014, and to 633 in 2015–2016 (Fig. 3a).

The percentage of tertian malaria cases without differentiation was around 1.3% during 2001–2013 and rose to 4.2% in 2014/15. The percentage of *Plasmodium malariae*, *Plasmodium ovale* and mixed malaria cases remained fairly stable over 16 years at around 2.5 (292/11.678), 2.8 (331/11.678) and 3.2% (374/11.678), respectively. In absolute numbers, all types of malaria, except knowlesi malaria, showed an increase in 2014 and 2015.

Over the whole observation period, 6 *P. knowlesi* cases were notified between 2012 and 2015. Five of those were native German persons who acquired malaria in Thailand: four were tourists and one visited friends and relatives (VFR). For one knowlesi malaria case, neither travel destination nor reasons for travel were indicated.

While for Africa as region of infection *P. falciparum* dominated other types by far (80.1%; 6192/7734), *P. vivax* was the most frequently reported species from Asia (64.1%; 430/671), South and Central America (55.6%; 114/205), and Oceania (68.4%; 65/95). From 2001 to 2013, before the marked increase in *P. vivax* notifications, the majority of vivax malaria cases were of German origin (55.0%; 527/958) or had migration background (21.5%; 206/958,), and 51/958 (5.3%) were recent refugees. Main countries of infection, accounting for more than half of the *P. vivax* cases, were India, Indonesia, Pakistan, Papua New Guinea, Brazil, and Cameroon.

In 2014–2016, 520 of 766 vivax malaria cases (67.9%) were recent refugees. 463 out of the 520 cases (89.0%) were men, 187 (36.1%) were children and adolescents under 18 years old; 415 (79.8%) were under 25 years old. The majority of those vivax cases originated from African countries (79.2%; 412/520), particularly from Eritrea (n = 378), Ethiopia (n = 12) and East Africa without specification (n = 7).

Mean malaria incidence in Eritrean refugees in Germany rose from 0.1% (yearly mean: 1/959.9) in 2001–2013 to 1.4% (yearly mean: 225/1684) in 2014–2015. Besides *vivax* malaria cases, 19 tertian malaria cases without differentiation, 11 *P. falciparum,* and 6 *P. ovale* infections were notified among Eritrean refugees during the observation period; 89.8% of all Eritrean refugees were male (442/492). Among refugees 21.2% of vivax malaria cases (71/335) originated from Asia in 2001–2016, particularly from Afghanistan (n = 32) and Pakistan (n = 36).

### Regions and countries in which infection was acquired

**Fig. 4:**
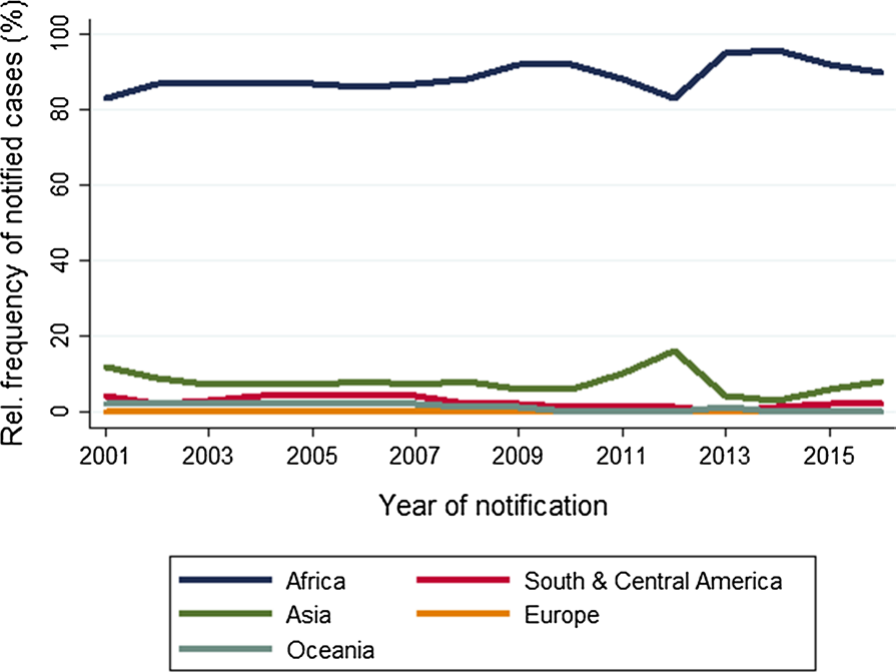
Malaria in Germany: relative frequency of continents of infection. Time series 2001–2016 (n = 8710)

Malaria was acquired on the African continent by 7734 out of 8710 (88.8%) cases, with indication of country/region of infection (Fig. 4); 95.4% (3803/3987) of cases with migration background and 82.6% (2983/3611) of native German cases had acquired malaria in Africa.

Overall, 54.7% (4718 of 8617 cases with indication of region) of cases had acquired malaria in West Africa. Infections from the Horn of Africa amounted to around 0.8% (54/6614) from 2001–2013, rose to 15.6% (210/1349) in 2014–2015 and dropped to 4.0% (26/654) in 2016 [2014–2016: 84.3% Eritrea (199/236), 12.7% (30/236) Ethiopia, 3.0% (7/236) Somalia]. In addition, in 2014–2016, 98 cases from East Africa without further specification and 28 cases from Sudan as region or country of infection were notified.

**Fig. 5:**
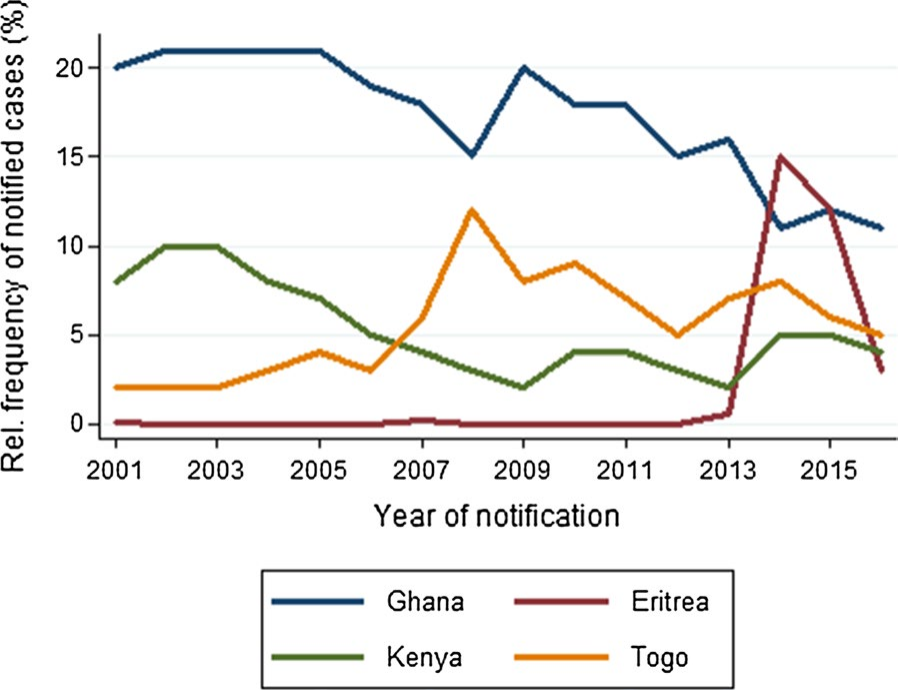
Malaria in Germany: relative frequency of malaria imported from Ghana, Eritrea, Kenya, and Togo. Time series 2001–2016 (n = 8710)

Overall, the five most frequently mentioned countries of infection were African countries (number of cases with information on country of infection: n = 8710): Ghana (17.2%; 1500/8710), Nigeria (14.5%; 1265/8710), Cameroon (9.7%; 849/8710), Kenya (5.6%; 487/8710), and Togo (5.1%; 448/8710). While Ghana and Kenya showed declining trends over 2001–2016, Togo was doubling case numbers in 2007–2016 compared to 2001–2006 (Fig. 5). Eritrea, which played almost no role as a country of infection until 2013, showed a 30-fold increase from 2013 to 2014/2015 with 97 and 85 cases, respectively (Fig. 5).

Out of 8710 cases with information on place of infection, 7.7% (n = 671/8710) acquired malaria in Asia. Some 102 cases had acquired malaria in Asia in 2001, while the annual average of cases was 37.9 (range 17–64) in subsequent years. The percentage of cases acquiring malaria in Asia was relatively stable for most of the time, amounting to an average of 7.4% (559/7551), except for a peak of 16.1% (62/386) in 2012, and a small increase in 2015 (Fig. 4). The rise in 2012 was mainly due to an increase of acquired infections in Pakistan (n = 33) and India (n = 18), and in 2015/2016 due to an increase in cases from Afghanistan (mean n = 18) and Pakistan (mean n = 17). While from 2001 to 2005 the majority of cases from Asia had acquired malaria in Southeast Asia [between 48.6% (17/35) in 2005 and 75.5% (77/102) in 2001, notably in Indonesia and Thailand], from 2010 onwards South Asia was the most important region of infection (Afghanistan, Pakistan, India) representing between 62.5% (10/16) in 2013 and 98.0% (50/51) in 2016 of infections from the Asian continent.

Two-hundred and five (2.4%; 205/8710) cases had acquired malaria in South and Central America and 1.1% (95/8710) in Oceania. Infections from those destinations showed an overall declining trend (Fig. 4). The most frequently reported countries of infection in Oceania and South and Central America were Papua New Guinea (n = 86), Brazil (n = 64), Dominican Republic (n = 34), and Venezuela (n = 23). An average of 13 annual cases were reported for Papua New Guinea in 2001–2004, which came down to 1–2 cases/year in 2009–2016.

Cases with countries of infection in Europe were making up 0.06% (5/8710) of all cases with information on place of infection. Two cases had *falciparum malaria*, 2 *vivax malaria* and 1 had a mixed infection. Two cases were reported with acquisition of malaria in France (without further specification, likely acquired in French overseas departments) and 3 in Germany. Nosocomial transmission was the probable cause for 2 cases with acquisition in Germany in 2007 and 2016. Both cases were women in their 30s, who were hospitalized at the same hospital ward as a patient with imported malaria caused by *P. falciparum*. Intense epidemiological investigations could not clarify the circumstances of transmission. For the remaining case information on the suspected mode of transmission was not provided.

### Use of chemoprophylaxis and reasons for travel

For calculation of the percentage of cases who had reportedly taken chemoprophylaxis only cases with reported countries or regions of infection, for which chemoprophylaxis was recommended were taken into account. Overall 1695 (24.3%) cases out of 6984 for whom this information was available had reportedly taken any type of chemoprophylaxis. For 2896 out of 9880 (29.3%) cases, information on chemoprophylaxis use was missing.

**Fig. 6:**
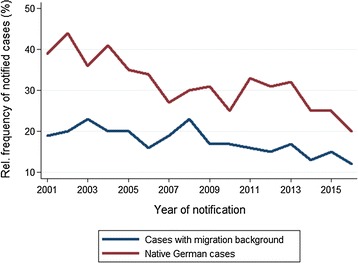
Malaria in Germany: chemoprophylaxis use in native German cases (n = 2896) vs. cases with migration background (n = 3750). Time series 2001–2016

Native German cases were significantly more likely to have taken chemoprophylaxis than cases with migration background [32.6% (943/2892) vs. 17.9% (669/3747); p < 0.001]. The percentage of cases taking chemoprophylaxis declined over the observation period in both groups (Fig. 6). Women were significantly more likely to have taken chemoprophylaxis than men [27.9% (589/2109) vs. 22.5% (1084/4809); p < 0.001]. Cases < 30 years of age among the native German sub group had significantly more frequently taken prophylaxis than cases aged 30 and older [41.7% (423/1014) vs. 27.7% (520/1878); p < 0.001]. This was not the case in the group of migrants (cases < 30 years of age: 16.1%, 160/997 vs. cases aged 30 and older: 18.5%, 509/2750; p = 0.082). Chemoprophylaxis did not differ significantly between persons who travelled as tourists compared to those travelling for work, education, and training [28.8% (358/1243) vs. 31.7% (374/1181); p = 0.124]. Cases who went for VFR were less likely to have taken chemoprophylaxis than others [22.4% (866/3867) vs. 30.2% (732/2424), p < 0.001].

**Fig. 7:**
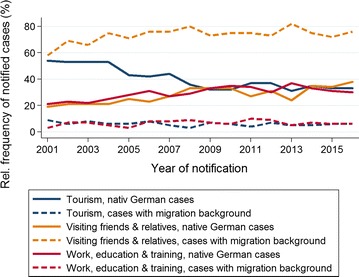
Malaria in Germany: reasons for travel among native German cases (n = 3761) vs. cases with migration background (n = 4357). Time series 2001–2016 (Multiple answers possible.)

By far the foremost reason for travelling to a malaria-endemic country among cases with migration background was VFR. For native German cases, the foremost reason for travel was tourism, in the years 2001–2007. From 2008, work, education and training and VFR became equally important (Fig. 7). Malaria incidence among air travellers to malaria-endemic countries has increased continuously from 0.52/1000 travellers in 2012 to 0.72/1000 travellers in 2016.

### Hospital admissions and fatalities

The overall hospital admission rate was fairly stable over the observation period (annual mean: 65.1%; 7601/11,678) and equal among the different groups. Children and patients with falciparum malaria or knowlesi malaria were significantly more frequently hospitalized than adults and patients with other types of malaria (p = 0.002 and p < 0.001, respectively). Hospitalization was reported for 68.7% (5641/8217) of falciparum malaria, 59.3% (1352/2279) of tertian malaria, 47.3% (138/292) of quartan malaria cases and for 5 out of 6 knowlesi cases. The mean lag time between symptom onset and start of treatment was slightly longer in cases with migration background and refugees (6.1 days) than in native German cases (5.6 days; p = 0.121).

During the observation period, 51 deaths due to malaria were notified, making up a yearly mean of 3.2 deaths (0.4%, 51/11,678; range 1–8). The largest number of fatal cases occurred in the year 2001. *Plasmodium falciparum* was causative for 43/51 (84.3%) of those fatalities, 4 were due to mixed infections. In 4 fatalities, *Plasmodium* species was not specified. Forty-two fatal cases were of German origin (82.4%; 42/51), 7 were residents with migration background (13.7%, 7/51), 1 was a recent refugee, and for 1 no information about the origin was indicated. Among the fatal cases, infection was most frequently acquired in Africa (88.2%; 45/51). Most frequently reported probable countries of infection were: Kenya (8/51), Ghana (6/51), Cameroon (5/51), Gambia (4/51) and Uganda, Togo and Senegal (3/51 each). Seven of the fatal cases, 6 native German cases and 1 person with migration background, had reportedly taken chemoprophylaxis. Three had taken mefloquine, one a atovaquone and proguanil combination medication, for three information on the regimen was unavailable. The delay between symptom onset and start of treatment could be calculated for 7645 non fatal cases and 24 fatalities. The mean was in both groups 5.8 days with an interquartile rage of 0–213 and 2–8 days, respectively. For treatment 28/51 (38.9%) fatal cases had received quinine, 17/51 (23.6%) doxycycline, 4/51 (5.6%) artesunate, 3/51 (4.2%) artemether, and 2/51 (2.8%) each atovaquone and mefloquine. Twenty (39.2%; 20/51) had received more than one drug and for 16/51 cases the information on the administered treatment was missing.

## Discussion

Time series analysis provides valuable data on trends of imported malaria cases in Germany. The decreasing trend in 2001–2009 did not continue. On the contrary, a sharp increase in malaria notifications was observed in 2014–2016 due to a high number of refugees from malaria-endemic countries arriving in Germany. At the same time, a mild increasing trend of malaria notifications over the past 10 years was observed among the German (non-refugee) population, along with changes in travel reasons and destinations and a decline in chemoprophylaxis use.

The largest part of the rise in *P. vivax* notifications was due to young male Eritrean refugees, as has been reported in 2014 from Sweden [8] and in 2016 from a German tropical medicine hospital [6]. Gender distribution in the German surveillance data showed a predominance of male cases for all malaria notifications during all years (men:women 2:1). This was attributed to differences in travel and prevention behaviour and to the composition of groups of people migrating to Germany [7]. The gender difference in 2014–2016 was more pronounced (men:women 3:1) and cases were on average significantly younger than in previous years. Why was there such a sudden increase of young men fleeing from Eritrea in 2014? According to the UN Human Rights Council [13] and Amnesty International [14] the main driver to leave Eritrea was the national conscription practice into military services which in practice amounts to forced labour [13, 14]. Since the law of the national service came into force in 1995, Eritreans have been fleeing their country, with large numbers residing in Sudan and Ethiopia [15]. According to the United Nations High Commissioner for Refugees (UNHCR) an intensified recruitment drive in 2014 and increasingly bad living conditions in the camps in neighbouring countries were causing a nearly 3-fold increase of Eritrean refugees in Europe in 2014, mainly in Germany, Sweden, Switzerland and the Netherlands [15]. The German Federal Office of Migration and Refugees reported a more than 23-fold increase of newly arriving Eritreans in 2014 and 2015, compared to the years 2001–2012 [11]. However, Eritreans accounted for 2.5% of asylum applications registered in 2015 in Germany only [11].

Most of the Eritrean cases presented with vivax malaria, only 5% were notified with falciparum malaria. According to the World Malaria Report 2016 [1], the predominant *Plasmodium* species in Eritrea and along the travel route (Ethiopia and Sudan) is *P. falciparum* (Eritrea: 75%; *P. vivax*: 25% [1]). As most Eritrean refugees travelled overland via Ethiopia and Sudan to Libya and then by boat to Italy [15], their travel time to arrival in Germany is longer than the usual incubation period for falciparum malaria. Sonden et al. (pers. comm.) report that a considerable number of cases arriving in Germany and Sweden had sought malaria treatment during migration, notably in Italy where national health services and non-governmental organizations provide medical care [16]. The high incidence of *P. vivax* hints at an accumulation of cases due to an increased probability of malaria acquisition in people who may have spent the nights outside during several months of travel, inadequately taken medication for relapse prevention (i.e., primaquine), due to unavailability or poor compliance, in addition to ongoing increased *P. vivax* transmission or outbreaks along the migration route (Sonden et al. pers. comm.).

Alongside refugees from Eritrea, there was an increase of malaria infections in refugees arriving from other countries of the Horn of Africa, Sudan, Pakistan, and Afghanistan. When plotting species distribution against time after withdrawing the population of recent refugees and cases without information on their origin, the rise of *P. vivax* cases in 2014 and 2015 was no longer visible (Fig. 3b). The refugee population, comprising many teenagers and young adults, might be more vulnerable and potentially less self-competent in attending medical care than adults who are acquainted with the German health care system. Clinicians must be aware of the possibility of malaria infection in refugees from the aforementioned countries, even if their arrival to Germany dates back several months. Relapses may occur frequently in inadequately treated patients and can occur when patients have resided in Germany for some time. Timely diagnosis is especially important, particularly as severe clinical pictures in this population were reported from different European countries probably due to delayed diagnosis and a generally impaired health status, presumably partly caused by the long and strenuous migration (Sonden et al. pers. comm.). This is an interesting observation, given that people from malaria endemic countries are likely to have acquired semi-immunity and should thus be partly protected against severe disease. The surveillance data do not provide any clinical information that could help to better understand this contradiction.

Overall, from 2001 to 2016, by far the most malaria infections were acquired in Africa with little variation in the most frequently reported countries (Ghana, Nigeria, Cameroon). The percentage of Asian countries of infection was low. It was fairly stable over the 15-year period (around 7%) with the exception of a 10% increase in 2012, which was mainly due to cases recently immigrated to Germany from Pakistan [17], and a rise in 2015 due to cases from Afghanistan and Pakistan. At the same time, groups who acquired malaria in Asia and countries of infection within Asia have changed. During the first half of the observation period the majority of cases were native Germans who had travelled as tourists to Southeast Asia, mainly to Indonesia and Thailand. From 2009 onwards, most cases were refugees or had migration background and were reported to have acquired malaria in Pakistan, India and Afghanistan. Southeast Asian countries were four times more frequently named as country of infection between 2001 and 2008 than between 2009 and 2016. This change may reflect an altering pattern of malaria epidemiology in Southeast Asia, or changes of travel destinations, or both. The risk for malaria in most regions in Southeast Asia is categorized as low, very low or non-existent. No regular chemoprophylaxis is recommended and carrying of standby emergency treatment for only some destinations [10]. The risk is still considered high for some regions in Indonesia [10]. But a decrease of malaria incidence in European travellers to Indonesia over the past decade, as described by Johansson Århem et al. [18], was also reflected in surveillance data for Indonesia and neighbouring Papua New Guinea.

Five knowlesi malaria infections acquired in Thailand were reported, and one additional without indication of travel destination. All those cases were reported between 2012 and 2016, mainly in tourists. It is likely that other *P. knowlesi* infections might have been misclassified as other malaria species, as correct microscopy diagnosis is complicated due to morphological similarity of the parasite with *P. falciparum* and *P. malariae* [19]. In addition, the same drugs as used for other types of malaria are effective in *P. knowlesi* cases [19]. It remains to be seen whether there will be more reports of *P. knowlesi* infections in the future as awareness of this *Plasmodium* species among microbiologists and clinicians rises and diagnostic tests improve.

An overall moderately rising trend of malaria notifications over the past 10 years was present among native German cases and residents with migration background. According to the German Federal Statistical Office, the number of air travel departures to malaria-endemic countries has increased by 10% between 2012 and 2016 [12]. Since 2006, the number of cases with migration background has exceeded native German cases. VFR was the foremost reported travel reason for cases with migration background during all 16 years of malaria surveillance. The largest sub-group of malaria cases overall were adult male residents with migration background who acquired falciparum malaria in Africa when VFR, sub-Sahara Africa and in particular West Africa being the regions with the highest risk of malaria acquisition. Among native German cases the picture has changed over the years. Tourism used to be the most important motive for travel, but its predominance was becoming less and less pronounced, while VFR, and work, education and training were becoming equally important reasons for travel. In the context of globalization, an overall growing mobility and an increasing number of people who are working and living abroad, and thus receiving friends and relatives for visits, might partly explain the overall increase of cases.

This data, showing an increasing trend of imported malaria in Germany, underlines the importance of pre-travel counselling, as malaria in travellers is at a large part preventable [20]. Schlagenhauf et al. [20] postulate that pre-travel consultations are associated with reduced morbidity of falciparum malaria in all traveller sub-groups. German surveillance data and notably data on countries or regions of infection are used for updating the malaria prophylaxis recommendations of the German society of tropical medicine and international health.

Inherently, malaria cases show a low percentage of chemoprophylaxis use, as compliant prophylaxis users are less likely to develop malaria. Nevertheless the reported share of chemoprophylaxis use was low (24%) compared to reports from other European countries [21, 22] and the USA [23], where it ranged from 28 to 40%. When calculating this percentage only cases with “yes” or “no” for chemoprophylaxis use were taken into account and not cases lacking information on this topic. Therefore, the percentage of chemoprophylaxis users was probably overestimated, as one can assume that cases with no information are more likely to have not taken chemoprophylaxis. Furthermore, the overall share of cases who did take chemoprophylaxis was continuously decreasing over the observation period. A similar trend is observed in other European countries [24] and the USA [23, 24]. The observed decrease was most pronounced among native German cases. Reasons can only be speculated about. Growing mobility and perceived easiness and affordability of travel to exotic destinations may alter a person’s attitude towards travel preparations and risk perception. Fear of side effects may also be a reason not to take chemoprophylaxis. An increasing trend of malaria incidence among air travellers to countries with known malaria transmission was observed between 2012 and 2016. Single incidence calculations might be quite inaccurate as neither overland (non-air) travel nor the varying malaria endemicity in different countries, nor seasonality were taken into account, but the overall trend is worrying [12].

The proportion of cases who took chemoprophylaxis was markedly lower among cases with migration background than among native German cases. This disparity is known [22, 24–26] and is mostly attributed to a lower risk perception of acquiring malaria and of suffering from severe malaria, and the lower uptake of pre-travel counselling by the population with migration background [25, 26]. This is particularly worrying since people whose families originate from malaria-endemic countries tend to use less mosquito protection measures, travel for longer periods and often to more remote areas than tourists and business travellers. In addition, this group of people is reported to seek medical attention in case of a febrile illness later upon return to Europe [25, 26]. Interestingly, despite residents with migration background taking less chemoprophylaxis, there were fewer fatal cases among them than among native German cases. This might be due to persisting partial immunity among cases originating from malaria-endemic countries. The surveillance data does not give information on attendance of pre-travel consultations. Awareness of the risk of malaria infection among travellers needs enhancement. Strategies aiming to increase uptake of and compliance to malaria chemoprophylaxis recommendations are needed, especially targeting those groups who were identified as taking only rarely chemoprophylaxis, e.g. residents with migration background. The present data reveal that malaria cases frequently report travel to high-risk areas, such as West Africa and therefore provide important travel medicine information for the training of physicians.

Notification data suffers from under-reporting. As a large number of cases was primarily diagnosed or transferred to large specialized centres for tropical medicine, which have well established notification mechanisms, a selection bias is unlikely to occur. Education of physicians to completely fill in the notification forms (e.g. country of infection) is needed to improve completeness of data. Misclassification of cases to different patient groups is possible. Recent refugees and residents with migration background might have less complete notification forms due to communication barriers. Therefore, more cases from those two groups might have been classified into the group with unknown origin than native German cases. This might have led to underestimation of a number of results, such as the problem of vivax malaria in refugee cases and the predominance of cases with migration background among all malaria cases. The notification form asks for the probable country of infection and does not include questions on exact travel destinations within the countries. When calculating percentages of chemoprophylaxis use all cases, who had travelled to countries with recommendation for chemoprophylaxis for at least part of the country were included. This might have led to an underestimation of chemoprophylaxis use. Changes over time in recommendations for prophylaxis could not be taken into account. Travel destinations for which in the past malaria prophylaxis was recommended, e.g. some countries in Southeast Asia, were thus excluded from the calculations. There is no reason to assume that those cases were systematically different from others.

## Conclusions

The steep rise in vivax malaria notifications in 2014 and 2015 was mainly due to newly arriving refugees from Eritrea but also due to refugees from other countries of the Horn of Africa and South Asia, predominantly young men. Clinicians should include malaria in their differential diagnosis in case of a febrile illness in this population even if arrival to Germany dates back several months.

Over the past 10 years malaria notifications among native Germans and residents with migration background showed an increasing trend. Use of chemoprophylaxis was insufficient in both groups and deteriorating. New strategies need to be found in order to increase compliance to chemoprophylaxis recommendations. The surveillance provides valuable data for the epidemiological assessment of imported malaria in Germany.
